# Drug-resistant *Mycobacterium tuberculosis*, Taiwan

**DOI:** 10.3201/eid1205.051688

**Published:** 2006-05

**Authors:** Ruwen Jou, Pei-Chun Chuang, Ying-Shun Wu, Jing-Jou Yan, Kwen-Tay Luh

**Affiliations:** * Center for Disease Control, Taipei, Taiwan, Republic of China;; † Chest Hospital, Tainan, Taiwan, Republic of China;; ‡ National Cheng Kung University Hospital, Tainan, Taiwan, Republic of China;; § National Association of Tuberculosis, Taipei, Taiwan, Republic of China

**Keywords:** Mycobacterium tuberculosis, drug resistance, Taiwan

**To the Editor:** Global surveillance of drug resistance has shown that a substantial proportion of tuberculosis (TB) patients are infected with drug-resistant *Mycobacterium tuberculosis* strains (*1*). Earlier hospital-based surveys have been undertaken in Taiwan, but these lacked systematic sampling and testing methods, which made interpreting results difficult. The combined treatment efficiency and the actual prevalence of drug resistance were unknown. Thus the Taiwan Center for Disease Control initiated the Taiwan Surveillance of Drug Resistance in Tuberculosis program in 2002.

A laboratory surveillance system was established and supervised by the national reference laboratory. The system includes 6 medical centers, 2 TB referral centers, and 1 regional hospital, distributed in 4 regions of Taiwan. The 9 laboratories provide services for healthcare facilities in their own and surrounding areas. Both the national reference laboratory and contract laboratories participated in an external quality proficiency test provided by the College of American Pathologists and the national reference laboratory. Performance was also assessed by the supranational reference laboratory in Antwerp, Belgium.

The population in the first year (2003) of the survey was 22,562,663, the number of confirmed TB cases was 15,042, the estimated incidence was 66.7 per 100,000 population, and the rate of notification of new positive sputum samples was 34.6% (*2*). A total of 3,699 isolates, ≈50% of *M. tuberculosis* strains isolated, underwent antimicrobial drug susceptibility testing in the system. Since clinical data were not available, only combined (primary plus acquired) drug resistance rates were analyzed. The survey showed that the combined drug resistance rates were 9.5% to isoniazid, 5.8% to ethambutol, 6.4% to rifampin, 9.6% to streptomycin, 20.0% to any drug, and 4.0% to multiple drugs. Resistance to any single drug was 12.3%, to any 2 drugs was 4.8%, to any 3 drugs was 2.2%, and to any 4 drugs was 0.7%. In the third global drug resistance surveillance report, the median prevalence of combined drug resistance was 6.6% to isoniazid, 1.3% to ethambutol, 2.2% to rifampin, 6.1% to streptomycin, 10.4% to any drug, and 1.7% to multiple drugs (*1*).

Available historical data from Taiwan are not directly comparable because of different sampling methods and because susceptibility testing methods have been applied in various hospital settings over time (Table), which limits our ability to monitor trends. The latest drug resistance rates obtained from Chest Hospital, a specialized TB referral hospital, showed that the combined drug resistance of any and multiple drugs were 27.6% and 15.8%, respectively, from January 2002 to June 2004 (unpub. data).

**Table:** Drug resistance patterns in Taiwan, 1960–2004

Hospital*	No. strains	Study period	Drug resistance (%)†							
			INH	EMB	RMP	SM	Any	MDR	Methods	Reference‡
Primary drug resistance										
A	162	1960–1962	13.4	–	–	11.7	22.2	–	Absolute concentration	(*6*)
B	154	1962	8.4	–	–	7.8	14.3	–		(*6*)
B	557	1971–1972	22.6	–	–	15.4	30.8	–	Resistance ratio on SM; absolute concentration on INH and EMB	(*6*)
B	1,914	1979–1982	8.4	0.1	0	9.2	17.9	–	Absolute concentration	(*6*)
B	1,924	1984–1988	6.8	0.4	0.2	5.0	9.9	–	Resistance ratio (1984–1986); absolute concentration (1986–1988)	(*6*)
B	1,935	1990–1995	9.2	0.7	1.5	5.7	12.3	1.2	Absolute concentration	(*6*)
B	249	1996	12.0	0.8	2.0	4.8	16.1	1.6	Absolute concentration	(*7*)
C	254	1996–1999	4.7	5.9	5.9	11.0	22.0	1.6	Proportion	(*8*)
D	456	2001–2002	14.9	2.6	3.3	11.4	20.6	2.4	BACTEC 960	(*9*)
E	190	2001–2002	11.1	5.8	2.1	5.3	16.8	2.1	Proportion	(*6*)
F	611	2002–2004	6.8	0.8	1.8	6.2	12.8	1.8	Proportion	NA
Acquired drug resistance										
B	200	1996	63.0	28.5	46.5	21.5	67.0	46.0	Absolute concentration	(*7*)
C	199	1996–1999	25.6	11.1	32.2	17.1	49.2	15.1	Proportion	(*8*)
B	183	2000–2001	37.7	10.9	25.1	17.5	42.6	24.6	Absolute concentration	(*10*)
D	57	2001–2002	31.6	15.8	17.5	19.3	36.8	15.8	BACTEC 960	(*9*)
E	62	2001–2002	54.8	33.9	45.2	17.7	64.5	45.2	Proportion	(*6*)
F	324	2002–2004	50.9	12.6	44.4	17.9	55.8	42.2	Proportion	NA
Combined drug resistance										
G	942	1982–1986	20.4	15.3	8.8	9.8	27.8	8.1	Proportion	(*11*)
A	651	1990–1992	14.7	10.3	10.6	11.2	22.6	8.3	Proportion	(*9*)
G	884	1992–1996	20.9	12.8	11.8	9.1	28.9	10.1	Proportion	(*11*)
B	1,091	1996	31.5	11.4	18.2	11.9	35.5	17.3	Absolute concentration	(*7*)
C	453	1996–1999	13.9	8.2	17.4	13.7	34.0	7.5	Proportion	(*8*)
H	693	1996–2000	35.9	15.7	13.4	28.6	52.4	11.4	Proportion	(*12*)
I	1,411	1998–2002	19.0	15.7	6.1	10.0	30.5	5.1	Proportion	(*13*)
D	513	2001–2002	16.8	4.1	4.9	12.3	22.4	3.9	BACTEC 960	(*9*)
E	252	2001–2002	21.8	12.7	12.7	8.3	28.6	12.8	Proportion	(*6*)
F	935	2002–2004	22.2	5.2	16.5	10.2	27.6	15.8	Proportion	NA

*A, Taipei Veterans General Hospital (medical center); B, Taiwan Provincial Chronic Disease Control Bureau (referral center); C, Taipei Municipal Chronic Disease Hospital (referral center); D, Changhua Christian Hospital (medical center); E, Buddhist Tzu Chi General Hospital (medical center); F, Chest Hospital (referral center); G, Chang–Gung Memorial Hospital (medical center); H, Kaohsiung Medical University Hospital (medical center); I, National Taiwan University Hospital (medical center). †INH, isoniazid; EMB, ethambutol; RMP, rifampin; SM, streptomycin; Any, resistance to any 1 of INH, EMB, RMP, or SM; MDR, resistance to at least INH and RMP. ‡NA, not available.

In Taiwan, isoniazid and rifampin were introduced in 1957 and 1978, respectively. Rifampin resistance was first seen in Taiwan in 1982. In recent decades, however, the rates of primary rifampin resistance have increased (Table), and primary resistance to multiple drugs has increased to 2.4% over time.

Based on patient data collected from Chest Hospital, multidrug resistance occurred in 42.2% of retreated TB patients, and 1.8% of multidrug-resistant isolates were found in new TB patients from January 2002 to June 2004 (unpub. data). In the third global drug resistance surveillance report, the median prevalence of multidrug resistance was 7.0% (highest 58.3%) among retreated cases and 1.1% (highest 14.2%) among new cases.

Significant declining trends were observed for any acquired resistance (67.0% to 42.6%, p<0.0001) and acquired multidrug resistance (46.0% to 24.6%, p<0.0001) at the Taiwan Provincial Chronic Disease Control Bureau from 1996 to 2001 (*3**,**4*). In addition, a decline in combined isoniazid resistance (43.1% to 16.4%, p < 0.0001), rifampin resistance (23.4% to 9.5%, p<0.0049), and multidrug resistance (18.2% to 7.8%, p<0.0113) was also reported from Kaohsiung Medical University Hospital from 1996 to 2000 (*5*). Taken together, data obtained from the Taiwan Surveillance of Drug Resistance in Tuberculosis and those reported previously show that rates of combined resistance to any drugs and multiple drugs has declined in Taiwan.

For retreated cases, the high acquired resistance rates indicated suboptimal initial treatment and insufficient case management of new patients, which raises a challenge to the National TB Control Programme in Taiwan. The direct observed treatment, short-course (DOTS) strategy has consequently been suggested to expand to all patients with newly diagnosed cases. The Taiwan Surveillance of Drug Resistance in Tuberculosis program will be extended to collect each patient's clinical and epidemiologic data, according to principles suggested in the guidelines prepared by the World Health Organization.
